# Understanding the Role of PknJ in *Mycobacterium tuberculosis*: Biochemical Characterization and Identification of Novel Substrate Pyruvate Kinase A

**DOI:** 10.1371/journal.pone.0010772

**Published:** 2010-05-24

**Authors:** Gunjan Arora, Andaleeb Sajid, Meetu Gupta, Asani Bhaduri, Pawan Kumar, Sharmila Basu-Modak, Yogendra Singh

**Affiliations:** 1 Institute of Genomics and Integrative Biology (CSIR), Delhi, India; 2 Department of Zoology, University of Delhi, Delhi, India; University of Hyderabad, India

## Abstract

Reversible protein phosphorylation is a prevalent signaling mechanism which modulates cellular metabolism in response to changing environmental conditions. In this study, we focus on previously uncharacterized *Mycobacterium tuberculosis* Ser/Thr protein kinase (STPK) PknJ, a putative transmembrane protein. PknJ is shown to possess autophosphorylation activity and is also found to be capable of carrying out phosphorylation on the artificial substrate myelin basic protein (MyBP). Previous studies have shown that the autophosphorylation activity of *M. tuberculosis* STPKs is dependent on the conserved residues in the activation loop. However, our results show that apart from the conventional conserved residues, additional residues in the activation loop may also play a crucial role in kinase activation. Further characterization of PknJ reveals that the kinase utilizes unusual ions (Ni^2+^, Co^2+^) as cofactors, thus hinting at a novel mechanism for PknJ activation. Additionally, as shown for other STPKs, we observe that PknJ possesses the capability to dimerize. In order to elucidate the signal transduction cascade emanating from PknJ, the *M. tuberculosis* membrane-associated protein fraction is treated with the active kinase and glycolytic enzyme Pyruvate kinase A (mtPykA) is identified as one of the potential substrates of PknJ. The phospholabel is found to be localized on serine and threonine residue(s), with Ser^37^ identified as one of the sites of phosphorylation. Since Pyk is known to catalyze the last step of glycolysis, our study shows that the fundamental pathways such as glycolysis can also be governed by STPK-mediated signaling.

## Introduction

The survival of a pathogen within the host in rather unfavorable conditions depends on its ability to manipulate its structural proteins and the regulatory components such as the proteins of the bacterial signal-transduction pathway which ensure that the structural factors are spatially and temporally regulated. A myriad of signaling molecules including some bacterial protein kinases have been studied as potential drug targets. Signaling pathways in *M. tuberculosis* are governed by 11 two-component systems, 11 eukaryotic type STPKs, one Ser/Thr phosphatase, one tyrosine kinase and two tyrosine phosphatases [1], [2]. Amongst the aforementioned signaling components, Ser/Thr protein kinases (STPKs) have gained considerable prominence in the past decade and PknB, PknG, PknH and PknJ have been patented as the drug targets [3]. Most of the *M. tuberculosis* STPKs are biochemically characterized and have been shown to possess important role in mycobacterial biology [4]–[12]. Mycobacterial STPKs have been suggested to influence diverse pathways and play significant roles in survival and metabolism of bacilli [13]–[30].

Although back in 1998, PknJ was predicted to be encoded in *M. tuberculosis* genome, work on this kinase has not progressed since there was no indication of associated signaling components [1]. Based on the sequence analysis of the kinase domain of *M. tuberculosis* STPKs, PknJ is positioned in the same clade as that of PknF and PknI [31]. These observations provide some insights into the evolution of PknJ; however, the functional significance of the kinase still remains largely unexplored. The present study focuses on the biochemical characterization of *M. tuberculosis* PknJ and identification of its substrates.

In parallel with other *M. tuberculosis* STPKs, PknJ also possesses N-terminal cytosolic domain and C-terminal extracellular domain. The C-terminal domain of STPKs is thought to sense environmental changes, which are communicated to the internal milieu through the N-terminal domain constituting the active site of the kinase [32]. Constructs comprising of the full length and cytosolic domain of PknJ were generated, overproduced and purified as active proteins. The active kinases displayed the requirement of some unusual cofactors, such as Ni^2+^ and Co^2+^, in addition to Mg^2+^ and Mn^2+^ which are required by many STPKs. The effect of metal ions on the kinase activity depends on the binding affinity of the enzyme to metal-ATP complex. Hence varying concentrations of Ni^2+^ and Co^2+^, *in vivo*, might play a regulatory role by affecting kinase activation and consequently the substrate phosphorylation. Further, we show that PknJ forms a dimer that might be important for the regulation of its kinase activity and ligand binding. Additionally, point mutations of the critical residues in PknJ were examined to verify the catalytic and activation mechanism as exhibited by other *M. tuberculosis* STPKs.

To explore the novel substrates of PknJ, we opt to analyze the *M. tuberculosis* membrane associated proteins. The analysis of membrane fraction is not only challenging but also provides an opportunity to explore the crucial membrane bound molecules, which can be utilized for better therapeutic and prophylactic interventions against tuberculosis [33]. Using this strategy, Pyruvate kinase A (mtPykA, Rv1617) is identified as a novel substrate of PknJ. Additionally, *M. tuberculosis* Serine/Threonine phosphatase (Mstp, Rv0018c) is shown to dephosphorylate both PknJ and mtPykA, thus proving that autophosphorylation and transphosphorylation of PknJ and mtPykA are reversibly regulated.

PknJ phosphorylates mtPykA on Ser^37^ as one of the phosphorylation sites. We suspect that phosphorylation of the Ser^37^ is dependent on the N-terminal arginine residue (pS/pT-4). The results establish a new phosphorylation motif: [R-X-X-X-S], analogous to eukaryotic protein kinase A (PKA) target motif: [RXXS/T] and [RXS/T] [34]. The motif predicts a number of new phosphorylation sites in known substrates of *M. tuberculosis* kinases. Pyk is historically known to catalyze the last step of glycolysis using phosphoenol pyruvate (PEP) and ADP as the substrates to produce pyruvate and ATP [35]. We have designed a novel assay to assess the mtPykA activity as a measure of ATP generation. Pyruvate kinase is a rate-limiting enzyme in glycolysis which also plays an important role in cell metabolism. Phosphorylation of mtPykA by a STPK further implicates a regulatory role played by STPKs in mycobacterial physiology.

## Results

### 1) Bioinformatic analysis of PknJ

**Figure 1:**
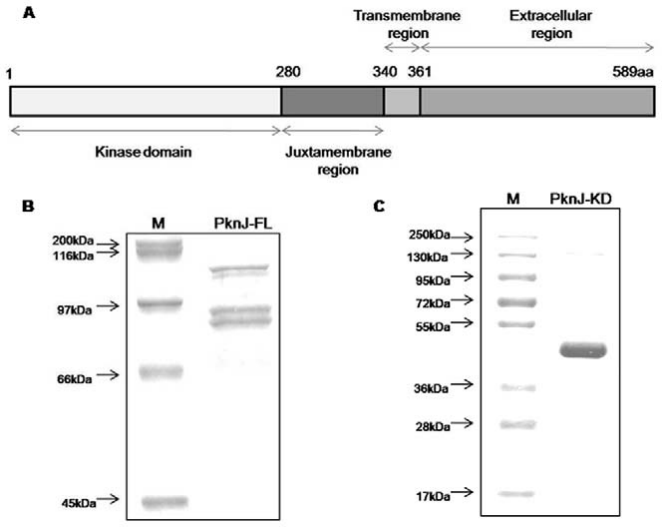
Domain architecture and protein purification of full length and kinase domain of *M. tuberculosis* PknJ (Rv2088). (A) A schematic representation of PknJ domain organization. The domains were predicted using TMHMM (http://www.cbs.dtu.dk/services/TMHMM/), HMMTOP (http://www.enzim.hu/hmmtop/) and DAS (http://www.sbc.su.se/~miklos/DAS/). (B) Full length PknJ was purified as GST-tagged protein of ∼93 kDa which migrates as duplet. Purified GST-PknJ-FL was run on 8% SDS-PAGE and stained with Coomassie Brilliant Blue. (C) Cytosolic kinase domain of PknJ containing 1–320 amino acids was purified as His_6_-tagged protein of 39 kDa which migrates at a higher size of ∼43 kDa. Purified His_6_-PknJ-KD was run on 12% SDS-PAGE and stained with Coomassie brilliant blue.

*M. tuberculosis* Rv2088 encodes for *pknJ*, the gene product of which is proposed to be a 589 amino acid long protein with a calculated molecular mass of 61.5 kDa and an estimated pI of 7.8. TMHMM, HMMTOP and DAS software were used to predict the topology of PknJ and on the basis of consensus derived from these softwares, we speculate that the kinase is a transmembrane protein with an intracellular N-terminal domain (1–343 amino acid) and an extracellular C-terminal domain (362–589 amino acid) (Fig. 1A). PknJ kinase domain shows considerable identity with other *Mycobacterium* STPKs (including maximum identity of 50% with PknF). The extracellular region of PknJ does not seem to resemble to any of the characterized proteins at sequence level. Given the unique nature of this domain, it may be involved in sensing signals which are exclusive to this kinase. STPKs are broadly categorized as Arg/Asp (RD) kinase family and non-RD kinase family proteins based on the presence of the RD-sequence in the catalytic loop [36]. Notably, the catalytic center of PknJ at the N-terminal domain carries the characteristic RD sequence. Further, STPKs conventionally constitute 12 conserved Hank's subdomains which are also located in the N-terminal domain of PknJ [37]. The Lys^43^ of PknJ was identified as the active site subdomain-II conserved lysine, known to be involved in orienting ATP α- and β-phosphates and making salt bridge with carboxyl group of subdomain-III glutamate residue of STPKs [37].

### 2) Over-expression and purification of PknJ full length and kinase domain

The PknJ full length protein was cloned in pProEx-HTc and pGEX-5x-3 expression vectors. While attempts to purify the recombinant full length kinase from *E. coli*-BL21 cells harboring pProEx-Htc-*pknJ* were unsuccessful even after varying the IPTG concentration (0.1 mM–1.0 mM) and induction temperatures between 16°C–37°C, *E. coli*-BL21 cells with pGEX-5x3-*pknJ* induced by 0.2 mM IPTG at 25°C for 1.5 hr yielded full length kinase (GST-PknJ-FL) at an approximate size of 93 kDa from soluble fraction (Fig. 1B). For the ease of purification and better yields, the cytosolic domain of PknJ (PknJ-KD) corresponding to residues 1–320 amino acid was cloned in pProEx-HTc, overproduced and purified as a His_6_-tagged protein. Extended kinase domain was taken as it has been reported that for kinase activity and stabilization, additional residues of juxtamembrane region are required. His_6_-PknJ-KD migrated at ∼43 kDa on SDS-PAGE (Fig. 1C), even though the expected size corresponded to 39 kDa. The aberrant migration can possibly be attributed to the presence of phosphorylated isoforms of PknJ-KD as reported for other mycobacterial kinases (PknD and PknE) [6], [7].

### 3) Protein kinase activity of PknJ and active site mutants

**Figure 2:**
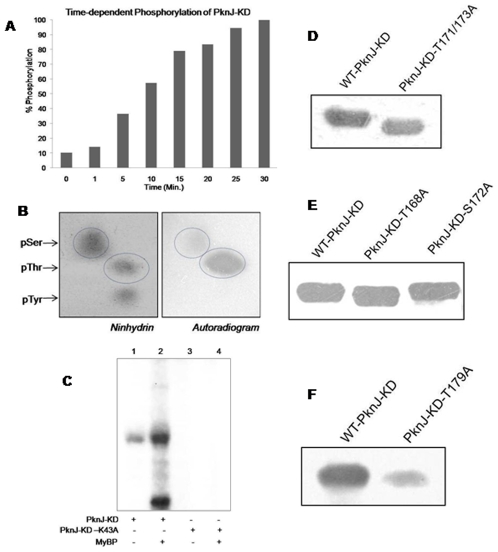
*In vitro* kinase assay of PknJ, Phosphoamino acid analysis and Immunoblotting of PknJ-KD and activation loop mutants. (A) Graph showing time-dependent autophosphorylation of PknJ-KD. After *in vitro* kinase assay with [γ-^32^P]ATP, gel was analyzed by PhosphorImaging and counts were calculated with MultiGauge (FujiFilm). Counts obtained after 30 min of reaction were taken as 100% signal and relative phosphorylation was estimated. Experiment was performed twice and the results indicate average of the two. (B) Analysis of phosphoamino acid content of autophosphorylated PknJ-KD. Amino acid standards, phosphoserine (pSer), phosphothreonine (pThr) and phosphotyrosine (pTyr) were added in the radiolabeled sample and visualized by ninhydrin staining (left panel) prior to autoradiography (right panel). The labeled pThr, pSer and their corresponding standards are encircled. (C) *In vitro* autophosphorylation of PknJ-KD (1 µg) and phosphotransfer on 5 µg Myelin basic protein (MyBP). Autophosphorylation deficient mutant PknJ-KD-K43A was used as negative control for auto- and transphosphorylation. The reactions were run on 12% SDS-PAGE and gel was autoradiographed after drying. As shown, transphosphorylation on MyBP was visible only in the presence of PknJ-KD, lane2. (D, E, F) 2 µg of *in vitro* phosphorylated native kinase along with its activation loop mutants (as indicated) were resolved on 10% SDS-PAGE, transferred onto nitrocellulose membrane and probed with anti-phosphothreonine antibody as described in experimental procedures. Autoradiograms are shown.

To demonstrate the kinase activity of PknJ, *in vitro* kinase assays were performed with GST-PknJ-FL and His_6_-PknJ-KD using [γ-^32^P]ATP as phosphate donor. Analysis of kinase assay by SDS-PAGE followed by phosphorimaging showed the presence of labeled proteins at approximately 93 kDa and 43 kDa corresponding to GST-PknJ-FL and His_6_-PknJ-KD respectively. Further autophosphorylation activity of PknJ-KD was examined in time-dependent manner to assess its efficiency. As shown in Fig. 2A, >50% autophosphorylation of PknJ-KD is achieved in less than 10 minutes. Transphosphorylation activity of GST-PknJ-FL and His_6_-PknJ-KD was evaluated by phosphorylation of artificial substrate myelin basic protein (MyBP) (Fig. 2C, Figure S1). Mutation of the Hank's subdomain II lysine residue to alanine (K43A) in His_6_-PknJ-KD resulted in the inactivation of the kinase, as observed by the loss of autophosphorylation and transphosphorylation signals on His_6_-PknJ-KD-K43A and MyBP respectively (Fig. 2C). These results verify that Rv2088 encodes for an active kinase and Lys^43^ represents the residue critical for catalyzing the phosphorylation.

Next we analyzed the nature of the phosphorylated amino acid residues in His_6_-PknJ-KD through phosphoamino acid analysis using two-dimensional thin layer electrophoresis (2D-TLE). After *in vitro* kinase assay in the presence of [γ-^32^P]ATP, His_6_-PknJ-KD was subjected to SDS-PAGE and transferred to Immobilon PVDF membrane followed by acid hydrolysis. As phosphorylation on serine, threonine and tyrosine is base labile and acid-resistant only these phosphoamino acids were detected [6]. Subsequently, PknJ-KD was observed to be autophosphorylated both on serine and threonine residues (Fig. 2B).

To identify the residues labeled during autophosphorylation, activation loop residues Thr^168^, Thr^171^, Ser^172^, Thr^173^ and Thr^179^ in PknJ (Figure S2) were mutated. Notably, threonine residues at similar position, Thr^171^ and Thr^173^ are localized in the activation loop of PknB and have been previously shown to be crucial for its activity [38]. Corresponding residues in PknJ were mutagenized to create double mutant T171A/T173A along with single mutants T168A, S172A and T179A and were compared with wild type protein. The immunoblotting of purified proteins after *in vitro* kinase assays with anti-phosphothreonine antibodies displayed comparable phosphorylation (Fig. 2D, 2E), except for T179A variant (Fig. 2F), which showed loss of phosphorylation signal. Hence, Thr^179^, present at the verge of activation loop, is probably one of the phosphorylation sites in PknJ. Consequently, these results also exhibit that PknJ is autophosphorylated not only on Thr^171^ and Thr^173^ but also on auxiliary residues.

### 4) Ionic regulation of PknJ autophosphorylation

**Figure 3:**
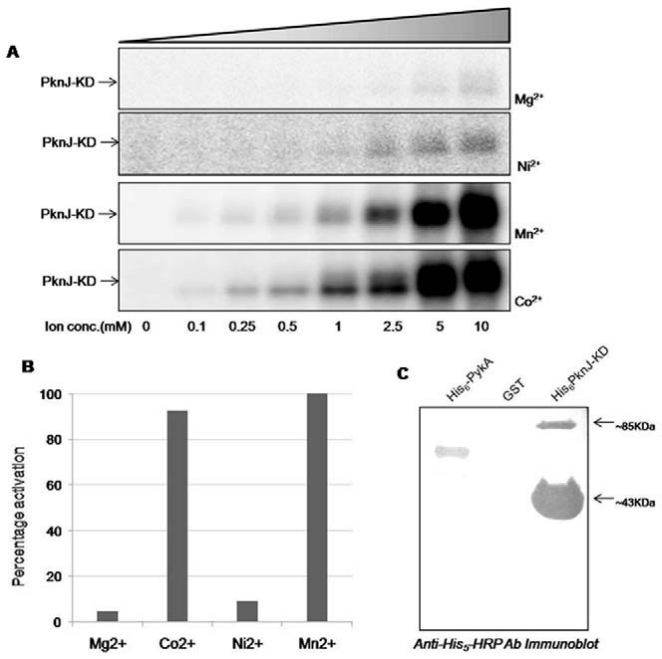
Regulation of PknJ-KD by metals-ions and dimer formation. (A) 1 µg PknJ-KD was incubated with increasing concentrations (0–10 mM) of different metal ions, individually in *in vitro* kinase assays. (B) Comparative analysis of PknJ-KD activation by MgCl_2_, MnCl_2_, CoCl_2_ and NiCl_2_ (10 mM each). Quantification of PhosphorImager units was done using ImageGauge software (Fujifilm) and converted to percentage activation. (C) To show PknJ-KD dimerization by immunoblotting, excess of PknJ-KD was resolved on 10% SDS-PAGE, transferred onto nitrocellulose membrane and probed with anti-His_5_HRP-conjugated antibody. His_6_-PykA and GST were taken as positive and negative controls, respectively.

Dependence on cofactors such as metal ion provides information on the structural and catalytic requirements for the enzymatic function of a given protein. While tyrosine kinases favor Mn^2+^-ATP as their cofactor, Ser/Thr protein kinases are thought to be dependent on Mg^2+^-ATP [39]. To determine the specific ion requirement of PknJ, various divalent cations were supplemented in the reaction buffer. Maximal kinase activity was observed in the presence of 10 mM Mn^2+^. Further, when Mg^2+^ was used as the cofactor, only marginal autophosphorylation was observed (Fig. 3A). Other than PknJ, the kinases PknB, PknD and PknI have also been shown to depend on Mn^2+^solely or in combination with Mg^2+^ for their activity [5], [6], [10]. To further elucidate the metal ion dependency of PknJ, Co^2+^, Ni^2+^, Zn^2+^ and Fe^2+^ cofactors were included in the kinase reactions individually. Interestingly, PknJ-KD was observed to be remarkably activated in the presence of Co^2+^ ions whereas, Ni^2+^ ions also displayed slight effect (Fig. 3A), while Fe^2+^ and Zn^2+^ had no effect on kinase activity (data not shown). Comparison of PknJ autophosphorylation activity by different ions reflects the marked difference with Mn^2+^ and Co^2+^ being the major effectors of kinase activity (Fig. 3A, Fig. 3B). These results indicate that the PknJ activity is dependent on various cofactors, some of them being rather exceptional for the mycobacterial STPKs.

### 5) Dimerization of PknJ

In eukaryotes, STPKs utilize dimerization as the widespread mechanism to regulate the kinase activity and ligand binding. Of the eleven kinases of *M. tuberculosis*, PknB, PknD and PknE are shown to form dimers, although the role of dimerization in modulating the kinase still remains to be established [40]–[42]. The sequence analysis of PknJ revealed the conservation of the predicted dimer-interface. Evaluation of the PknJ orthologs present in different species of *Mycobacterium* showed that PknJ has retained the conserved dimerization interface, when compared with the crystal structures of PknE, PknD and PknB.

During the purification of PknJ-KD and its mutants, a band at approximately 85 kDa was observed on SDS-PAGE, corresponding to twice the size of PknJ-KD. The same protein band was observed to be phosphorylated during PknJ kinase assays which could not be visualized in the absence of divalent cations or when PknJ-KD-K43A was used for kinase assay (data not shown), though the band was visible in coomassie stained gel. Immunoblotting using anti-Penta-His HRP-conjugated antibody directed against the recombinant His_6_ tag of PknJ-KD confirmed that the band corresponds to PknJ (Fig. 3C). Mass Spectrophotometric analysis of the protein present in coomassie stained band in both wild type and PknJ-KD-K43A also validated it to be PknJ.

Based on structural and functional studies on PknE and PknD dimer, we mutated analogous conserved dimer interface residue His^78^ in PknJ-KD to Ala [41], [42]. Mutation of His^78^ resulted in loss of autophosphorylation activity as compared to native protein (Figure S3), though electrophoretic analysis of this protein on coommassie stained SDS-PAGE gel still shows the dimer-band (Figure S4).The integrity of this band was also verified by western blot with anti anti-Penta-His HRP-conjugated antibody (data not shown). These results suggest the involvement of multiple residues in PknJ dimerization.

### 6) Identification of mycobacterial proteins as probable substrates of PknJ

**Figure 4:**
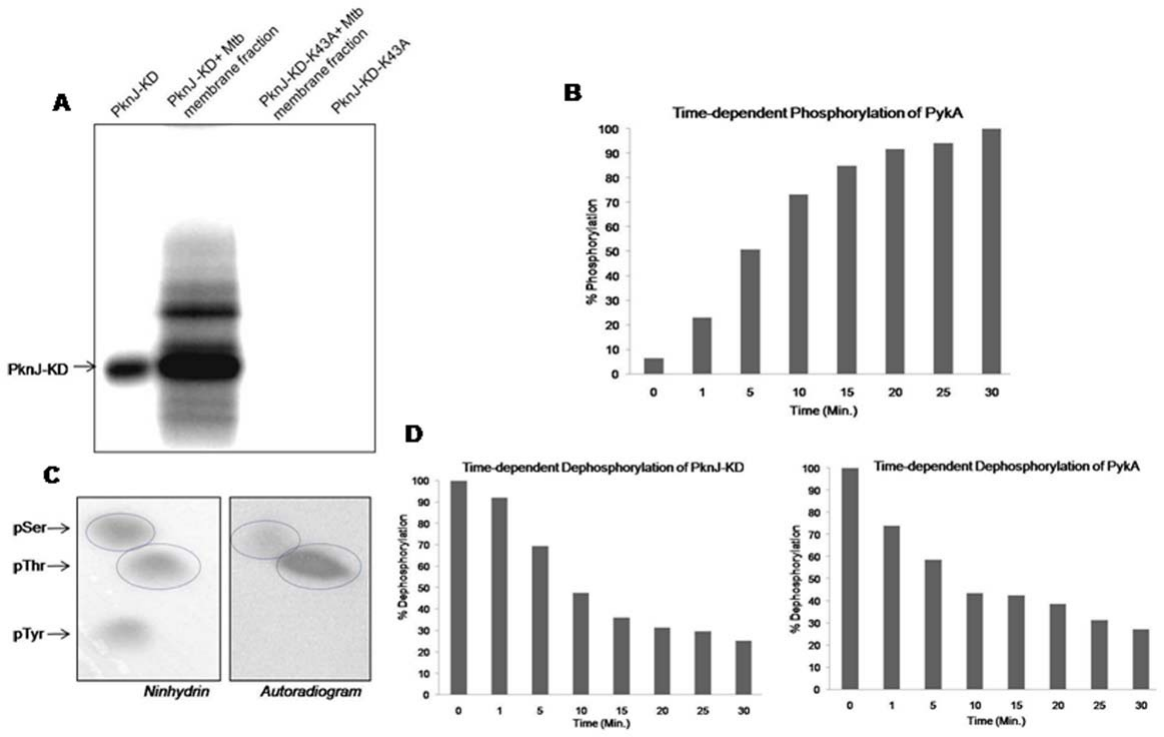
*In vitro* phosphorylation of membrane-associated protein fraction and mtPykA by PknJ-KD. (A) 20 µg purified fraction of membrane-associated proteins was incubated with 2 µg of PknJ-KD or PknJ-KD-K43A, in an *in vitro* kinase assay. Samples were separated by 10% SDS-PAGE and autoradiographed on PhosphorImager. (B) Graph showing time-dependent phosphorylation of mtPykA by PknJ-KD. 1 µg of PknJ-KD was incubated with 2 µg of mtPykA with increasing time-points (0–30minutes) in *in vitro* kinase assay. Phosphorylation was evaluated as before. Experiment was performed twice and the results indicate average of the two. (C) Phosphoamino acid content of mtPykA phosphorylated by PknJ-KD was assessed as discussed earlier. (D) Time-dependent dephosphorylation of *in vitro* phosphorylated PknJ-KD (left panel) and mtPykA (right panel) by Mstp_cat_. Extent of dephosphorylation was measured by adding 500 ng of purified Mstp_cat_ to a reaction mixture containing phosphorylated PknJ-KD and mtPykA for increasing time-points. Dephosphorylation was evaluated as discussed before with signal intensity at 0 minute taken as maximum. Experiment was performed twice and the results indicate average of the two.

Mycobacterial membrane-associated proteins are proposed to participate in cell-cell interactions, ion transport and cell signaling [43]. We tried to establish the involvement of membrane-associated proteins in STPK mediated signaling. To identify the putative endogenous substrates, we incubated PknJ-KD with the cell extracts of *M. tuberculosis*, representing the proteins associated with membrane fractions in the presence of [γ-^32^P]ATP. Several phosphorylated protein bands were observed in the autoradiogram of one-dimensional SDS-PAGE (Fig. 4A). PknJ-KD-K43A, having no kinase activity, was used as the negative control. Three of the maximally phosphorylated protein bands were identified by MALDI-TOF/TOF as probable substrates of PknJ of which two were identified as the novel substrates of mycobacterial STPKs. Pyruvate kinase A (mtPykA, Rv1617) and probable lactate dehydrogenase (Lldd2, Rv1872c) were the newly identified substrates, while GroEl2 (Rv0440) has been previously suggested to be phosphorylated by *M. tuberculosis* kinases [22]. Prompted by these observations, we tried to establish the detailed phosphorylation status of mtPykA by PknJ.

### 7) *In vitro* phosphorylation of mtPykA

In an attempt to verify the above observation, kinase assay was set up using recombinant mtPykA and PknJ. The gene encoding *pykA* (Rv1617) was cloned into *E. coli* expression vector and expressed as a recombinant His_6_ -tagged-PykA and GST-tagged-PykA proteins of 53 kDa and 76 kDa respectively.

MtPykA, GST- as well as His_6_-tagged proteins, were confirmed to be phosphorylated by PknJ-KD while PknJ-KD-K43A showed no phosphotransfer (data not shown). Further experiments proved authenticity of phosphorylation on mtPykA and not on the recombinant tag. Time-dependent phosphorylation of mtPykA by PknJ-KD was performed to investigate efficiency of phosphotransfer. Substantial phosphorylation of mtPykA within a few minutes of reaction indicated that mtPykA was promptly phosphorylated by the kinase (Fig. 4B). MtPykA phosphorylation was also confirmed by GST-PknJ-FL (Figure S5).

To determine the phosphorylated residues, 2D-TLE was performed on mtPykA after the kinase reaction with PknJ-KD. Both serine and threonine residues were found to be phosphorylated with the major label localized on the threonine residue(s) (Fig. 4C). Sequence analysis shows that mtPykA contains 23 serine and 29 threonine residues. The presence of both serine and threonine signals on autoradiogram entails that PknJ phosphorylates mtPykA on multiple sites.

### 8) Dephosphorylation of PknJ and mtPykA by mycobacterial Ser/Thr phosphatase, Mstp

The mechanism of reversible phosphorylation executed through kinases and phosphatases is a mode of altering biochemical or structural properties of protein and is utilized for all primary biological functions. Mycobacterium encodes for one PP2C-class Ser/Thr phosphatase, Mstp (Rv0018c), which has been previously shown to act on STPKs and their substrates [38], [44]. After establishing that PknJ-KD and mtPykA were phosphorylated on serine and threonine residues, we assayed for their dephosphorylation by Mstp_cat_ in a time dependent manner (Fig. 4D). Mstp_cat_ hydrolyzed the phosphate moiety of Ser/Thr residues of both PknJ and mtPykA as observed by decrease in PhosphorImager counts, with ∼80% signal lost after 30minutes of phosphatase addition.

### 9) Identification of Ser^37^ as potential phosphorylation site of mtPykA

**Figure 5:**
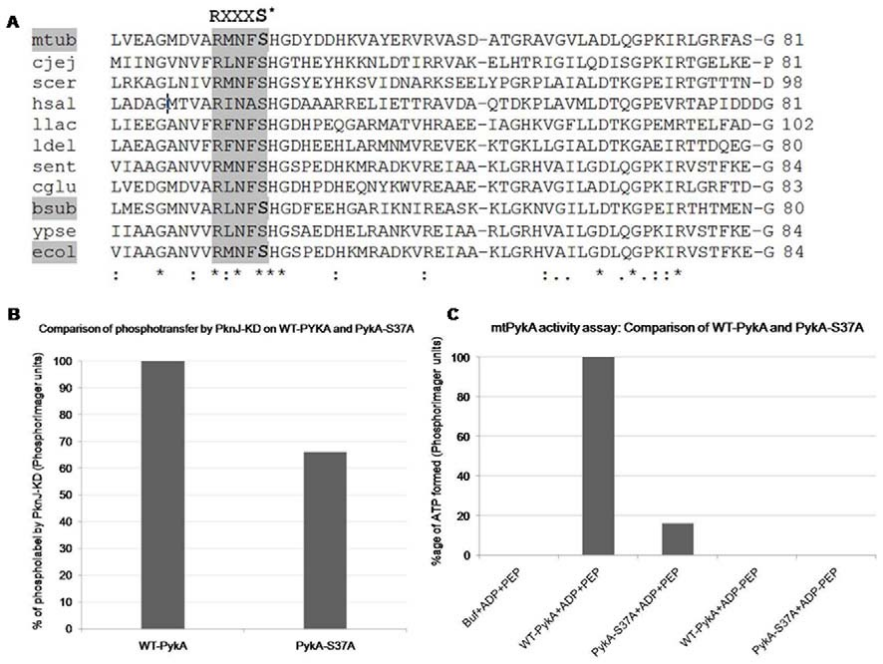
Importance of mtPykA Ser^37^ as a target site of PknJ-KD and as a critical residue for mtPykA activity. (A) Multiple sequence alignment of Pyruvate kinase from various microbial species using t-coffee server (http://www.ebi.ac.uk/Tools/t-coffee/index.html). The conserved RXXXS* motif is highlighted with S* being the phosphorylated residue in *M. tuberculosis*, *E. coli* and *B. subtilis* is shaded grey. Pyk sequences were taken from following species: *Mycobacterium tuberculosis* (mtub.), *Campylobacter jejuni* (cjej.), *Saccharomyces cerevisiae* (scer.), *Halobacterium salinarum* (hsal.), *Lactococcus lactis* (llac.), *Lactobacillus delbrueckii* (ldel.), *Salmonella enterica* (sent.), *Corynebacterium glutamicum* (cglu.), *bacillus subtilis* (bsub.*), Yersinia pseudotuberculosis* (ypse.) *and Escherichia coli* (ecol.). (B) Loss of phospholabel on PykA-S37A with respect to WT-PykA. Similar concentrations of WT-PykA and PykA-S37A (2 µg) were incubated with 1 µg PknJ-KD in the presence of [γ-32P]ATP. Image was analyzed by ImageGauge as discussed before. (C) Comparison of WT-PykA and PykA-S37A in terms of ATP generation. Activity assays were performed with [α-^32^P]ADP and analyzed by ImageGauge.

Phosphoamino acid analysis revealed that mtPykA was phosphorylated on serine and threonine residues. To interpret the consequence of phosphorylation, it is fundamental to identify the site of phosphorylation on the substrate. Earlier reports on phosphoproteome analysis suggested that the Pyruvate kinase homologs in *E. coli* and *B. subtilis* are phosphorylated [45], [46]. Importantly, in *E. coli* and *B. subtilis*, Pyk homologs are phosphorylated on Ser^36^ residue. In *M. tuberculosis*, PykA Ser^37^ corresponds to Ser^36^ of *E. coli* PykF and *B. subtilis* Pyk (Fig. 5A). With the purpose of analyzing Ser^37^ as the possible site of phosphorylation, mtPykA Ser^37^ was converted to alanine by site directed mutagenesis. *In vitro* kinase assays with the mutant and the wild type protein resulted in the partial loss of phosphorylation on PykA-S37A mutant as compared to the WT-PykA (Fig. 5B) suggesting that Ser^37^ is one of the target sites of phosphorylation. Since a complete loss of phospholabeling was not observed, the presence of additional phosphorylatable sites is proposed.

Further the analysis of the *B. subtilis* Pyk, *E. coli* PykF and mtPykA implied the presence of conserved arginine, three residues upstream of phosphorylated serine outlining the motif RXXXS. The motif is present in pyruvate kinase from diverse species (Fig. 5A) and thus notifies the conservation of phosphorylation site of pyruvate kinase in multiple species. The motif is quite similar to Human PKA motif; further bioinformatic analysis reveals the presence of such motif in many reported STPK substrates (File S1). As proof of principle, we found Thr^77^ of Rv0019c and Thr^343^ of FtsZ, which are reported to be phosphorylated by PknA also belong to RXS/T and RXXS/T PKA-motif [14].

### 10) Role of Ser^37^ residue in mtPykA activity

In glycolysis, PykA utilizes phosphoenol pyruvate (PEP) and ADP to form Pyruvate and ATP. Thus, we employed WT-PykA and PykA-S37A to generate [α-^32^P]ATP using [α-^32^P]ADP and excess of PEP and analyzed by a TLC-based assay (Figure S6). Interestingly, WT-PykA could generate ∼10fold more ATP than its mutagenized counterpart (Fig. 5C), hence indicating towards Ser^37^ being an important residue for mtPykA activity. No ATP was observed in the controls which lack either mtPykA or PEP, showing that the observed effect was indeed due to mtPykA. The reaction was coupled to LDH assay to avoid accumulation of pyruvate and subsequent inhibition of mtPykA by its reaction product.

Next, we tried to perform resin-bound phosphorylation of WT-PykA and PykA-S37A using GST-PknJ-FL, to assess the effect of phosphorylation on mtPykA activity. We could not observe any change in the activity of phosphorylated or unphosphorylated enzymes. It may be attributed to the inefficient phosphotransfer on the immobilized- substrates by GST-PknJ-FL.

## Discussion

Present study emphasizes on the biochemical characterization of *Mycobacterium tuberculosis* Ser/Thr Protein Kinase PknJ and the identification of the glycolytic enzyme Pyruvate kinase A (mtPykA) as its substrate. PknJ is identified as an active kinase that requires Lys^43^ residue of subdomain II for its activity. PknJ is activated only in the presence of certain divalent cations such as Mn^2+^, Co^2+^, Mg^2+^ and Ni^2+^, with Mn^2+^and Co^2+^ preferred as opposed to the other two. Additionally, there is no effect of Zn^2+^ and Fe^2+^ ions on PknJ activity, indicating specificity of PknJ towards selective transition metals ions. Mn^2+^ is also reported to be the principle ion required for the activation of PknB, PknD and PknI but none of the kinases are known to get activated by Co^2+^ and Ni^2+^. Nickel and Cobalt play important role in *M. tuberculosis* survival in macrophages [47]. Urease activity which was shown to be critical for *M. tuberculosis* survival in phagosome is dependent on Nickel, while Vitamin B12 biosynthesis which is a Cobalt dependent process is important for *M. tuberculosis* survival in macrophages [47]. *M. tuberculosis* maintains the balance between metal mediated toxicity and supply of essential metal ions for its survival through dual Nickel-Cobalt sensors [47]. One aspect of Ni^2+^/Co^2+^ dependent regulation of PknJ can be the downstream substrate phosphorylation while interplay between kinase and metal sensor or their activating enzymes is also possible.

The cytosolic segment of the kinase, PknJ-KD, undergoes autophosphorylation on Ser and Thr residues. Site-directed mutants of two conserved Thr residues, PknJ-KD-T171/173A do not show complete loss of activity as shown for PknB [38]. This suggests a strong possibility of involvement of multiple residues in the activation process of PknJ. An important mechanism in the activation and regulation of sensor kinases is dimerization. A large number of Ser/Thr kinases are stated to form dimers, although the importance of dimerization for *M. tuberculosis* kinases is only beginning to unravel with few reports hypothesizing on the possible role of PknB, PknD and PknE dimerization. Even in the reducing conditions of SDS-PAGE, PknJ forms stable dimer, which is also shown by variants PknJ-KD-K43A and PknJ-KD-H78A. As reported for PknD-KD, mutation of dimerization interface residue His^78^ in PknJ-KD resulted in loss of autophosphorylation suggesting dimerization as an additional mechanism in allosteric regulation of kinase [41]. Further structural studies of PknJ will help in determining the conservation of structural arrangement of dimer interface among STPKs.

Signaling through transmembrane kinases is essentially carried out through the proteins phosphorylated by the kinases under specific conditions. Hence, a number of strategies are applied to identify the substrates of kinases and their possible regulation. We tried to identify the proteins phosphorylated by PknJ using the *M. tuberculosis* membrane-associated protein fraction. Using the specified approach, mtPykA, LLDD2 and GroEL2 were identified as the most prominently phosphorylated proteins by PknJ. GroEL2, which is vital for mycobacterial growth, has been suggested as the substrate of STPKs [22]. Thus, it can be speculated that chaperones are also controlled by multiple STPKs.

Previous work on mycobacterial PykA shows that in the absence of functional PykA, *M. bovis* loses its ability to use glycerol as carbon source [48]. MtPykA has multiple functions and affects central metabolism. The recent knockout study shows the impact of mtPykA in fatty acid and lipid biosynthesis and β-oxidation pathways [49]. The expression of a number of vital genes was observed to be affected in absence of PykA, showing that mtPykA is important for the cell but not indispensable.

We have ascertained that recombinant mtPykA gets phosphorylated by PknJ on both Ser and Thr residues. We have also identified Ser^37^ as one of the phosphorylated sites. Notably, Ser^37^ is a conserved residue found in Pyk homologs of other bacterial genera. Although MtPykA structure is unknown, Ser^37^ site in Pyk homologs of certain species is known to be critical for enzyme action [50]. In an assay using [α-^32^P]ADP and PEP as substrates to measure mtPykA activity resulting in generation of pyruvate and [α-^32^P]ATP, we observed the decreased ATP formation in mtPykA-S37A mutants with respect to native mtPykA, thus showing the regulation of mtPykA activity by this site.

Other than its structural and biochemical role, Ser^37^ as a phosphorylation site of mtPykA has evolutionary importance too. In eukaryotes, PykA is shown to be phosphorylated on a typical motif (RXXS/T) by Protein Kinase A (PKA) [51]–[53]. In case of mtPykA, Ser^37^ belongs to a slightly altered motif RXXXS. MtPykA has two homologs in *E. coli* (PykA and PykF) and one in *B. subtilis*. Incidentally, *B. subtilis* Pyk and *E. coli* PykF were identified to be phosphorylated at Ser^36^ residue in phosphoproteome analysis which also belongs to the RXXXS motif. These observations indicate the conservation of the phosphorylation motif in PykA from prokaryotes to eukaryotes. This is also supported by proposition projected in *E. coli* where 40% of phosphoproteins are conserved in eukaryotes, in contrast to ∼14% of the non-phosphorylated proteins and several phosphorylation sites being conserved from *Archaea* to humans [45].

Significant differences in activity of phosphorylated and unphosphorylated WT-PykA and PykA-S37A were not observed in terms of ATP generation (data not shown). It may be due to the inefficient phosphorylation of the resin bound mtPykA by GST-PknJ-FL. It is important to mention the fact that many glycolytic enzymes are known to possess the so-called moonlighting functions. Similarly, pyruvate kinase is proposed to perform nucleoside diphosphate kinase (Ndk) -like activity in *E. coli* under anaerobic conditions [54]. In *M. smegmatis*, the pyruvate kinase has been shown to interact with NDK and manipulate its activity such that it is only able to generate GTP [55]. In addition, Pyk is also involved in fatty acid and amino acid metabolisms by generating pyruvate as a precursor for many metabolites. Thus, there exists a possibility that mtPykA phosphorylation may affect the functions other than glycolysis.

In summary, we report that mycobacterial PknJ is a functional protein kinase that possesses some unusual activation requirements and autophosphorylation sites. PknJ is projected to phosphorylate mtPykA on multiple sites involving both serine and threonine residues. Ser^37^ is the influential residue for mtPykA which aids in ATP and pyruvate generation and is also identified as one of the phosphorylation site.

## Materials and Methods

### 1) Bacterial strains and growth conditions

*E. coli* strain DH5α (Novagen) was used for cloning and BL21 (DE3) (Stratagene) for the expression of recombinant proteins. *E. coli* cells were grown and maintained with constant shaking (220 rpm) at 37°C in LB medium supplemented with 100 µg/ml ampicillin and/or 40 µg/ml kanamycin, when needed. *M. tuberculosis H37Rv* was grown in Middlebrook 7H9 broth supplemented with 0.5% glycerol and 10% ADC at 37°C with shaking at 220 rpm for 3–4 weeks. Solid media included LB-Agar in case of *E. coli* and 7H10 agar containing 0.5% glycerol and 10% OADC supplement in case of *M. tuberculosis H37Rv*.

### 2) Cloning, expression and purification of PknJ and its mutants

The *pknj* gene, its fragments coding for PknJ-kinase domain (PknJ-KD) and juxta membrane region from residues 1–320 amino acids were PCR amplified using *M. tuberculosis H37Rv* genomic DNA as a template and forward and reverse primers (Table 1), containing XhoI and NotI (Roche) restriction sites for cloning into expression vector pGEX-5X-3 (GE Healthcare Bio-Sciences) and NotI and XhoI for cloning into expression vector pProEx-HTc (Invitrogen). The amplified products were digested with restriction enzymes and ligated into vectors pGEX-5X-3 and pProEx-HTc, previously digested with the same enzymes to yield plasmids mentioned in table 2. The pProEx-HTc-PknJ-KD construct was utilized for generating K43A, T171A/T173A, T168A, S172A, T179A and H78A mutations. pProEx-HTc-PknJ-KD was subjected to site-directed mutagenesis using QuikChange XL Site-Directed Mutagenesis Kit (Stratagene) and primer pairs carrying desired mutations (Table 1). The integrity of all constructs was confirmed by DNA sequencing.

**Table 1:** Details of primers used in the study.

S.No	Primer name	**Sequence (5'-3')	Description of Restriction site/mutation
1	PknJ-Htc FP	GGTTGATGGGCGGCCGCGTGGCCCACGAGTTGAGTG	NotI
2	PknJ-KD RP	ATGCTGTCACCACGGCGAACCTCGAGCTGAGAACCTGGC	XhoI
3	PknJ-HTc RP	GGATGTAGCTCGAGATGCCGATGACCGCCTGCTGGCCG	XhoI
4	PknJ-pGEX FP	GTACCATCTCGAGTGTGGCCCACGAGTTGAGTGCGG	XhoI
5	PknJ-pGEX RP	ATCATAGCGGCCGCTCAGCCGGGTATCTTGGCGAGGA	NotI
6	T171A/T173A FP	CGACACGGACTGGCGTCCGCCGGTTCGGTGCTGGCC	T171A/T173A
7	T171A/T173A RP	GGCCAGCACACCGAACCGGCGGACGCCAGTCCCGTGTCG	T171A/T173A
8	K43A FP	CGAAGCCTTGGCAGTCCTTGC	K43A
9	K43A RP	GCAAGGACTGCCAAGGCTTCG	K43A
10	T168A FP	GCGCGTGCGCTCGGCGACGCGGGACTGACGTCCACCGGT	T168A
11	T168A RP	ACCGGTGGACGTCAGTCCCGCGTCGCCGAGCGCACGCGC	T168A
12	S172A FP	GGCGACACGGGACTGACGGCCACCGGTTCGGTGCTGG	S172A
13	S172A RP	CCAGCACCGAACCGGTGGCCGTCAGTCCCGTGTCGCC	S172A
14	T179A FP	CCGGTTCGGTGCTGGCCGCGTTGGCCTATGCTGCG	T179A
15	T179A RP	CGCAGCATAGGCCAACGCGGCCAGCACCGAACCGG	T179A
16	H78A FP	CCCAACATCGTGGCGGTTGCTCAGCGCGGCCAGTTG	H78A
17	H78A RP	CAACTGGCCGCGCTGAGCAACCGCCACGATGTTGGG	H78A
18	Rv1617 FP	GCATACGGATCCTAGTTACGAGACGCGGGAAAATCGTC	BamHI
19	Rv1617 RP	TGATAGACTCGAGCTAGACGTCATCTTCCCCGATCCG	XhoI
20	S37A FP	GTCGCCCGAATGAACTTCGCCCACGGCGACTACGACG	S37A
21	S37A RP	CGTCGTAGTCGCCGTGGGCGAAGTTCATTCGGGCGAC	S37A

**The sites for restriction enzymes and point mutations are underlined.

**Table 2:** List of plasmids used in this study.

	Plasmid	Description	Reference
1	pGEX-5X-3	*E. coli* vector utilized to generate GST fusion proteins	GE Healthcare
2	pGEX-5X-3-PknJ-FL	pGEX-5X-3 derivative used to purify GST tagged Rv2088 (PknJ)	This work
3	pGEX-5X-3-PykA	pGEX-5X-3 derivative used to purify GST tagged Rv1617 (mtPykA)	This work
4	pProExHTc	*E. coli* vector used to generate His_6_-tag fusion proteins	Invitrogen
5	pProExHTc-PknJ-KD	pProExHTc derivative used to purify His_6_-tagged PknJ-KD containing cytosolic domain of PknJ	This work
6	pProExHTc-PknJ-KD-K43A	pProExHTc derivative used to purify His_6_ tagged PknJ-KD carrying K43A mutation	This work
7	pProExHTc-PknJ-KD-T171A/T173A	pProExHTc derivative used to purify His_6_ tagged PknJ-KD carrying T171A/T173A mutations	This work
8	pProExHTc-PknJ-KD-T168A	pProExHTc derivative used to purify His_6_ tagged PknJ-KD carrying T168A mutation	This work
9	pProExHTc-PknJ-KD-S172A	pProExHTc derivative used to purify His_6_ tagged PknJ-KD carrying S172A mutation	This work
10	pProExHTc-PknJ-KD-T179A	pProExHTc derivative used to purify His_6_ tagged PknJ-KD carrying T179A mutation	This work
11	pProExHTc-PknJ-KD-H78A	pProExHTc derivative used to purify His_6_ tagged PknJ-KD carrying H78A mutation	This work
12	pProExHTc-PykA	pProExHTc derivative used to purify His_6_ tagged Rv1617(mtPykA)	This work
13	pProExHTc-PykA-S37A	pProExHTc derivative used to purify His_6_ tagged mtPykA carrying S37A mutation	This work
14	pProExHTc-Mstp_cat_	pProExHTc derivative used to purify His_6_ tagged Mstp_cat_ containing cytosolic domain of Mstp	Ref no.[16]

*E. coli* BL21 (DE3) cells were transformed with pGEX-5X-3-PknJ-FL, pProEx-HTc-PknJ-FL and pProEx-HTc-PknJ-KD plasmids expressing full length or catalytic domain respectively. For over-expression, recombinant *E. coli* strains harboring the pGEX-5X-3-PknJ-FL was used to inoculate 1 L of LB medium supplemented with ampicillin followed by incubation at 37°C with shaking (220 rpm) until A_600_ reached 0.8. IPTG was added to a final concentration of 0.2 mM and growth was continued for additional 2 hr at 25°C. Cells were harvested and stored at −80°C. Pellets were thawed on ice and resuspended in cell lysis buffer A (50 mM Tris-Cl pH [8.0], 300 mM NaCl, 1 mM DTT, 1 mM EDTA, 1X Protease inhibitor cocktail (Roche) and 1 mM PMSF) and lysed by sonication. The cell lysates were centrifuged at 14 K rpm, 4°C for 20 min. The supernatant containing recombinant protein was collected and incubated with glutathione sepharose 4B affinity resin (GE Healthcare Bio-Sciences) pre-equilibrated with buffer A. After extensive washings with buffer A, resin was further washed with high salt concentration buffer (50 mM Tris-Cl pH [8.0], 1 M NaCl, 1 mM DTT, 1 mM EDTA, 1 mM PMSF and 10% glycerol). Elution was carried out in elution buffer (50 mM Tris-Cl [pH 8.5], 10% glycerol, 150 mM NaCl and 15 mM glutathione). Fractions were run on 10% SDS-PAGE and analyzed by coomassie brilliant blue staining. Pure fractions were dialyzed (20 mM Tris-Cl [pH 8.0], 10% glycerol and 150 mM NaCl), aliquoted and stored at −80°C.

The recombinant His_6_-tagged proteins harboring wild type and mutant PknJ, PknJ-KD, PknJ-KD-K43A, PknJ-KD-T171/173A, PknJ-KD-T168A, PknJ-KD-S172A, PknJ-KD-T179A and PknJ-KD-H78A were essentially purified as described above with minor alterations. For His_6_-tagged proteins, induction with IPTG was carried out for 12 hr at 15°C. After sonication with buffer B (50 mM Tris-Cl [pH 8.5], 300 mM NaCl, 5 mM β-mercaptoethanol, 1X Protease inhibitor cocktail (Roche) and 1 mM PMSF), sonicate was centrifuged at 14 K rpm, 4°C for 20 min. Supernatant obtained after centrifugation was incubated with Ni^2+^-nitrilotriacetic acid resin (Qiagen) previously equilibrated with buffer B. The soluble proteins were processed as described in the previous section. Washings and elution were carried out as described previously [16]. Buffers used were (50 mM Tris-Cl [pH 8.5], 1 M NaCl, 5 mM β-mercaptoethanol, 20 mM Imidazole, 10% Glycerol and 1 mM PMSF) and (50 mM Tris-Cl [pH 8.5], 150 mM NaCl, 10% Glycerol and 200 mM Imidazole) for washings and elution respectively.

### 3) Cloning, Expression and Purification of WT-PykA and PykA-S37A

The genes encoding for *pykA* were PCR amplified using *M. tuberculosis* genomic DNA, using primer pair Rv1617 FP and Rv1617 RP (Table 1). The amplicons thus generated were digested with BamHI and XhoI restriction enzymes respectively and ligated into pProEx-HTc, pET-28c (Novagen) and pGEX-5X-3 vectors previously digested with similar enzymes. The plasmids thus constructed are mentioned in table 2. Serine mutation (S37A) in pProEx-HTc-*pykA* was generated using QuikChange XL Site-Directed Mutagenesis Kit (Stratagene) using primer pairs carrying desired mutations (Table 1). The recombinant proteins were essentially purified as described above with minor alterations. GST-tagged protein was induced with 1 mM IPTG for 3 hr at 37°C while for His_6_-tagged proteins, induction with IPTG was carried out for 12 hr at 15°C. After sonication with buffer A/B (as described above) WT-PykA and PykA-S37A were solubilized from inclusion bodies using solubilization buffer (1.5% N-lauryl sarcosine, 25 mM Triethanolamine, 2% Triton X-100, 50 mM Tris-Cl [pH 8.5], 300 mM NaCl, 5 mM β-mercaptoethanol/1 mM DTT, 1X Protease inhibitor cocktail (Roche) and 1 mM PMSF). The supernatant obtained after centrifugation (14 K rpm for 20 min at 4°C) was incubated with Ni^2+^-nitrilotriacetic acid resin (Qiagen) and further processed as described in previous section.

### 4) *In vitro* kinase reaction and phosphoamino acid analysis

*In vitro* kinase assays were performed with 1–2 µg of GST-PknJ-FL, PknJ-KD and its mutants (K43A, T171/173A, H78A) added separately in 1X kinase buffer (20 mM PIPES (pH 7.2), 5 mM MgCl_2_, 5 mM MnCl_2_) alone or with 2–4 µg of desired substrate protein (WT-PykA, PykA-S37A, MyBP, GST) and 20 µg mycobacterial membrane associated fractions. Reactions were started with the addition of 10 µCi [γ-^32^P]ATP (BRIT, Hyderabad, India) and were incubated at 25°C for 20 min or 0–30 min for time-dependent phosphorylation assay. The reactions were terminated by the addition of 5X-SDS sample buffer and a subsequent boiling for 5 min at 100°C. Samples were resolved by appropriate percentage of SDS-PAGE. Gels were fixed in 30% methanol and visualized by FLA2000 PhosphorImager (Fujifilm).

Experiments concerning variations of different metal-ions were performed with 1 µg of PknJ-KD incubated with 20 mM PIPES (pH 7.2) and increasing concentrations (0–10 mM) of MgCl_2_, MnCl_2_, CoCl_2_, NiCl_2_, ZnCl_2_ and FeCl_2_ each, at 25°C for 20minutes. For evaluation of PknJ-KD activation by ions was essentially performed as above with 10 mM of MgCl_2_, MnCl_2_, CoCl_2_ and NiCl_2_ each. The image analyses were done with help of ImageGauge software (Fujifilm) and PhosphorImager units were plotted for comparison.

### 5) Immunoblotting

To check the involvement of activation loop residues Thr^168^, Thr^171^, Ser^172^, Thr^173^ and Thr^179^, WT-PknJ-KD and its mutants were used for immunoblotting. Equal amounts of *E. coli* purified proteins were run on SDS-PAGE after *in vitro* kinase assay (with cold ATP) and transferred onto nitrocellulose membrane (Bio-Rad). After overnight blocking of membrane with 3% BSA in PBST (Phosphate buffer saline [pH 7.2], 0.1% Tween 20), the blot was incubated for 1 hr at room temperature with antibodies dissolved in PBST at 1∶10,000 dilution, directed against phosphothreonine (Invitrogen). Followed by five washes the blot was incubated for 1 hr at room temperature with anti-rabbit HRP polyclonal antibody dissolved in PBST at 1∶10,000 dilution. After five washes the blots were developed using SuperSignal^R^ West Pico Chemiluminescent Substrate kit (Pierce Protein Research Products) according to manufacturer's instructions.

The dimerization status of PknJ-KD, PknJ-KD-K43A and PknJ-KD-H78A was estimated by immunoblotting of the purified kinases with anti-His_5_HRP-conjugated antibodies which recognize the his_6_-tag associated with the kinase. Blotting was performed as explained above except that after incubation with primary antibody, the blots were developed using diamino benzidine/H_2_O_2_ as substrate for HRP following the five washes with PBST. Glutathione-S-Transferase (GST) was used as negative control and His_6_-tagged mtPykA was the positive control for the assay.

### 6) Time-dependent dephosphorylation of PknJ and mtPykA

Cytosolic domain of Rv0018c (Mstp_cat_) which was derived earlier was used for dephosphorylation assays [16]. Time dependent dephosphorylation of phosphorylated PknJ and mtPykA was achieved by supplementing Mstp_cat_ (500 ng) after kinase reaction at 25°C. The reactions were stopped with SDS-sample buffer followed by boiling and resolved on 10% SDS-PAGE. The signals were visualized by PhosphorImager.

### 7) Phosphoamino acid analysis

After *in vitro* kinase reaction, phosphorylated PknJ-KD and mtPykA were separated by 10% SDS-PAGE and transferred onto Immobilon PVDF membrane (Millipore). The phosphorylated proteins were detected by autoradiography and the bands corresponding to [γ-^32^P] labeled substrates were excised and hydrolyzed in 6 M HCl for 1 hr at 110°C. The hydrolyzed samples containing liberated phosphoamino acids were lyophilized and re-dissolved for phosphoamino acid analysis by 2D TLE as described [56].

### 8) Preparation of mycobacterial membrane-associated protein fraction

*M. tuberculosis H37Rv* cells were cultured in 50 ml of 7H9 broth medium for 14 days at 37°C with gentle shaking. Culture was pelleted and washed with phosphate-buffered saline, pH 7.2 and inactivated by UV-irradiation for 30 minutes. The cells were further harvested at 8000 rpm for 10 minutes. The supernatant was discarded and pellet was resuspended in 10 ml of solubilization buffer (50 mM Tris-HCl, pH 7.4, 150 mM NaCl, 1 mM EDTA, 5 mM MgCl_2_, 1 mM PMSF and 1X protease inhibitor cocktail) and the cells were lysed by sonication (Misonix Sonicator 3000).The suspension was centrifuged at 15000 rpm for 30 minutes at 4°C. The pellet was further dissolved in 100 mM sodium carbonate buffer and ultracentrifuged (Beckman Coultor Optima Max ultracentrifuge) in MLA 130 rotor 150,000×*g* at 4°C for 1 hr. The recovered membrane pellets were resuspended further in 2 ml buffer (7 M urea, 2 M thiourea, 1% SDS and 10% glycerol) for 2 hr and were concentrated/dialyzed against buffer containing 50 mM Tris pH 7.4, 150 mM NaCl and 10% glycerol using 10 KDa Amicon centrifugal filters (Millipore). This fraction was further used in PknJ kinase assay to identify the phosphorylated proteins. The reaction was terminated by addition of SDS sample buffer and the reactants were resolved by SDS PAGE and visualized using PhosphorImager.

### 9) Activity assays of WT-PykA and PykA-S37A

[α-^32^P]ADP was generated using [α-^32^P]ATP (BRIT, Hyderabad, India) in an ATPase assay with Rv1747 as described [57]. [α-^32^P]ADP was separated on cellulose TLC-plate (Merck biosciences) extracted and redissolved in MilliQ water. 5 µg of His_6_-tagged PykA and PykA-S37A were incubated with purified [α-^32^P]ADP in reaction buffer (8 mM PEP (Sigma), 200 ng lactate dehydrogenase (Sigma), 0.5 mM NADH (Sigma), 50 mM Tris-Cl, 10 mM MgCl_2_, 150 mM KCl) at 37°C for 2 hr. Reaction with [α-^32^P]ATP were used as positive control and those without enzyme or PEP were the negative controls. The reactions were stopped by heating the samples at 80°C for 15 minutes. Equal volumes of all the samples were loaded on cellulose TLC-plates and separated in one-dimension with 0.6 M KH_2_PO_4_ pH 3.75 as the solvent system. Formation of [α-^32^P]ATP was analyzed by PhosphorImager.

## Supporting Information

Figure S1 In vitro autophosphorylation of full length kinase (PknJ-FL) (2 µg) and phosphotransfer on 5 µg Myelin basic protein (MyBP). The reactions were run on 12% SDS-PAGE and gel was autoradiographed after drying. Due to very low in vitro activity of PknJ-FL, marginal phosphotransfer by observed on MyBP (lane2).
(2.79 MB TIF)

Figure S2 Multiple sequence alignment of activation loop of M. tuberculosis STPKs was done using t-coffee server (http://www.ebi.ac.uk/Tools/t-coffee/index.html). The conserved ser and thr residues of PknJ are highlighted.
(2.79 MB TIF)

Figure S3 In vitro kinase assay of PknJ-KD and PknJ-KD-H78A. Autoradiogram shows loss of kinase activity in dimer-interface mutant as compared to native kinase, though the dimer band is visible in both proteins.
(2.79 MB TIF)

Figure S4 Coomassie stained SDS-PAGE showing the presence of dimer band in PknJ-KD, its kinase dead mutant PknJ-KD-K43A and dimer-interface mutant PknJ-KD-H78A. Proteins were loaded in excess to show clear band of dimer.
(2.79 MB TIF)

Figure S5 In vitro phosphorylation of mtPykA by PknJ-FL. 2 µg of kinase was incubated with 3 µg mtPykA. The reactions were run on 12% SDS-PAGE and gel was autoradiographed after drying.
(2.79 MB TIF)

Figure S6 Activity assay of WT-PykA and PykA-S37A. Autoradiograph of cellulose-TLC is shown. Lane1: [α-32P]ATP +PEP + buffer control, Lane2: [α-32P]ADP + PEP+ buffer control, Lane3: [α-32P]ADP + PEP + WT-PykA, Lane4: [α-32P]ADP + PEP + PykA-S37A, Lane5: [α-32P]ADP + WT-PykA-PEP control, Lane6: [α-32P]ADP + PykA-S37A-PEP control. Loss of ATP generation is evident in case of PykA-S37A as compared to WT-PykA.
(2.79 MB TIF)

File S1 List of previously reported selective substrates of M. tuberculosis STPK containing the putative phosphorylation motifs RXXXS/T, RXXS/T and RXS/T.
(0.03 MB DOC)
