# Supplementary figures and images for: Understanding the Role of PknJ in Mycobacterium tuberculosis: Biochemical Characterization and Identification of Novel Substrate Pyruvate Kinase A
Source: PLoS One. 2010 May 24;5(5):e10772. doi: 10.1371/journal.pone.0010772 (PMC2875399; doi:10.1371/journal.pone.0010772)

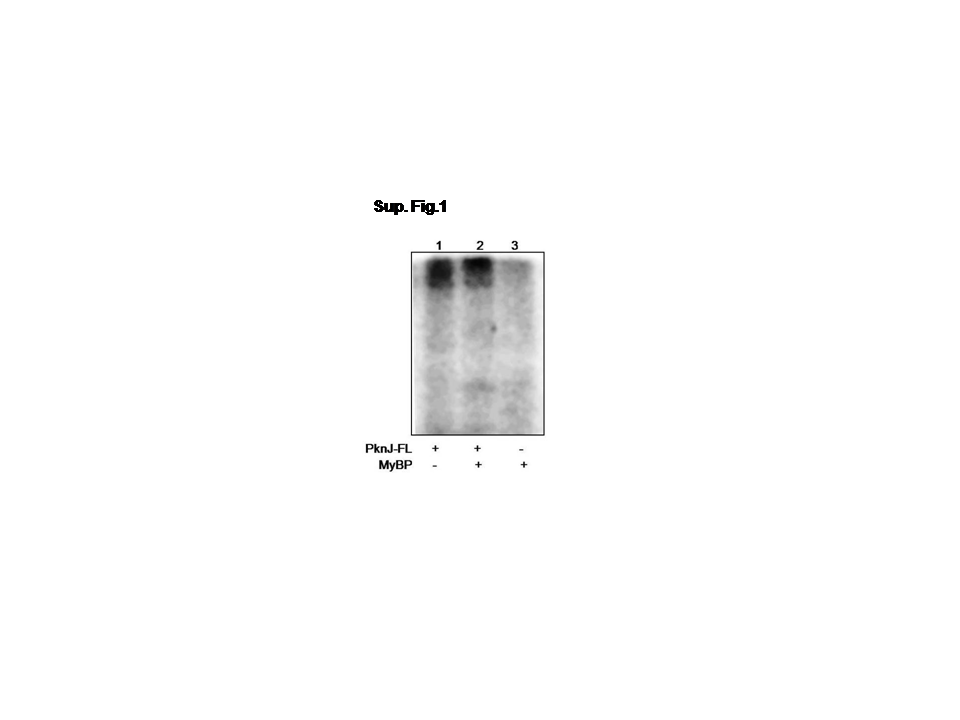

Supplement: Figure S1 — In vitro autophosphorylation of full length kinase (PknJ-FL) (2 µg) and phosphotransfer on 5 µg Myelin basic protein (MyBP). The reactions were run on 12% SDS-PAGE and gel was autoradiographed after drying. Due to very low in vitro activity of PknJ-FL, marginal phosphotransfer by observed on MyBP (lane2). (2.79 MB TIF) [file pone.0010772.s001.tif]

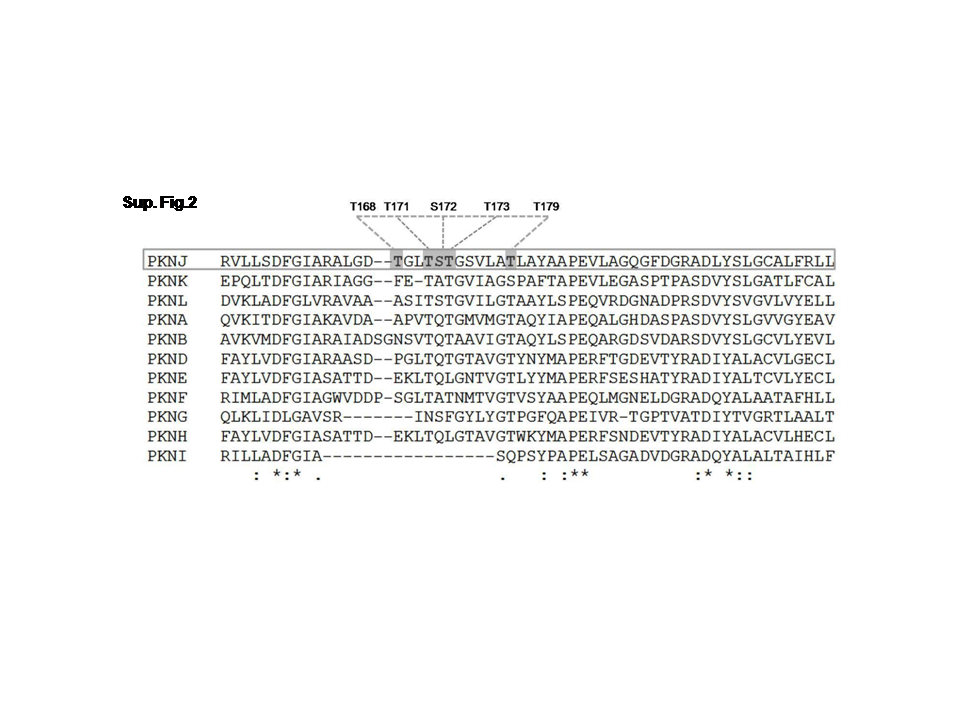

Supplement: Figure S2 — Multiple sequence alignment of activation loop of M. tuberculosis STPKs was done using t-coffee server (http://www.ebi.ac.uk/Tools/t-coffee/index.html). The conserved ser and thr residues of PknJ are highlighted. (2.79 MB TIF) [file pone.0010772.s002.tif]

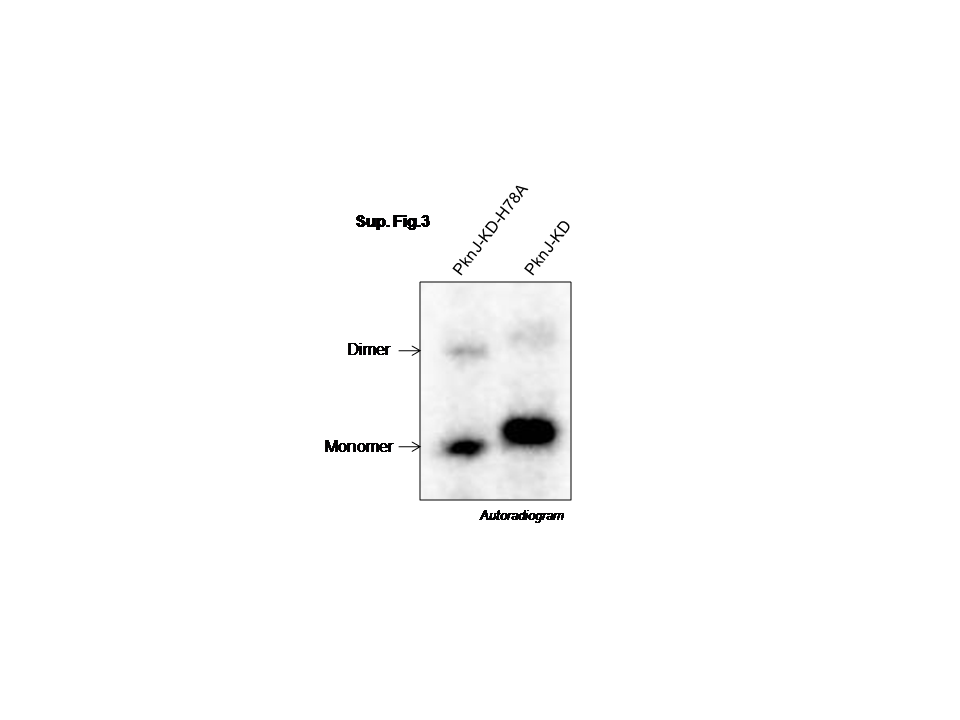

Supplement: Figure S3 — In vitro kinase assay of PknJ-KD and PknJ-KD-H78A. Autoradiogram shows loss of kinase activity in dimer-interface mutant as compared to native kinase, though the dimer band is visible in both proteins. (2.79 MB TIF) [file pone.0010772.s003.tif]

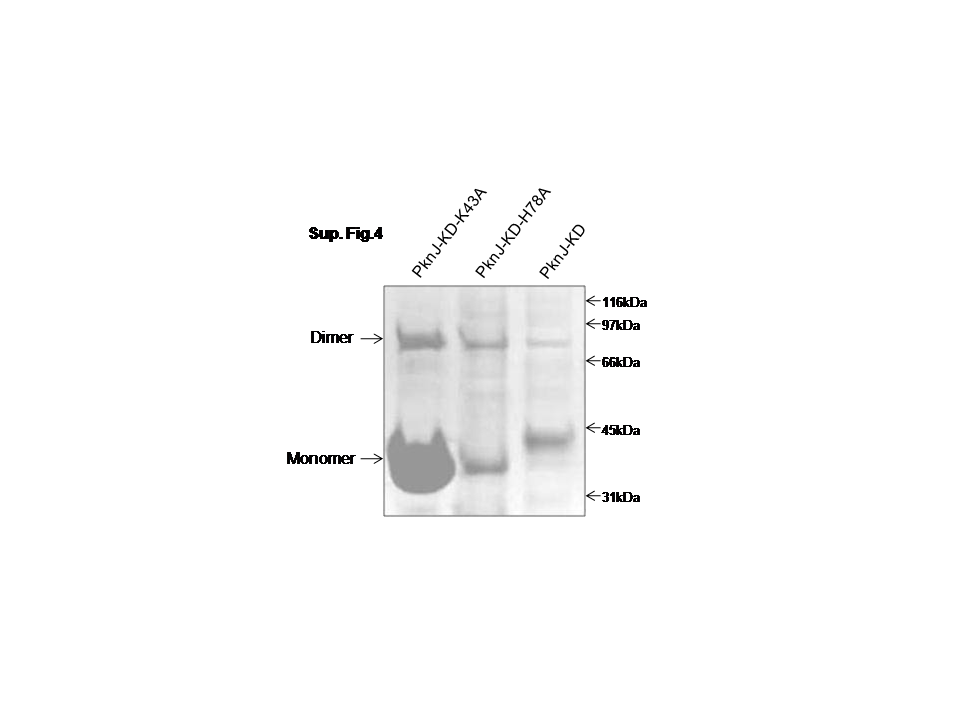

Supplement: Figure S4 — Coomassie stained SDS-PAGE showing the presence of dimer band in PknJ-KD, its kinase dead mutant PknJ-KD-K43A and dimer-interface mutant PknJ-KD-H78A. Proteins were loaded in excess to show clear band of dimer. (2.79 MB TIF) [file pone.0010772.s004.tif]

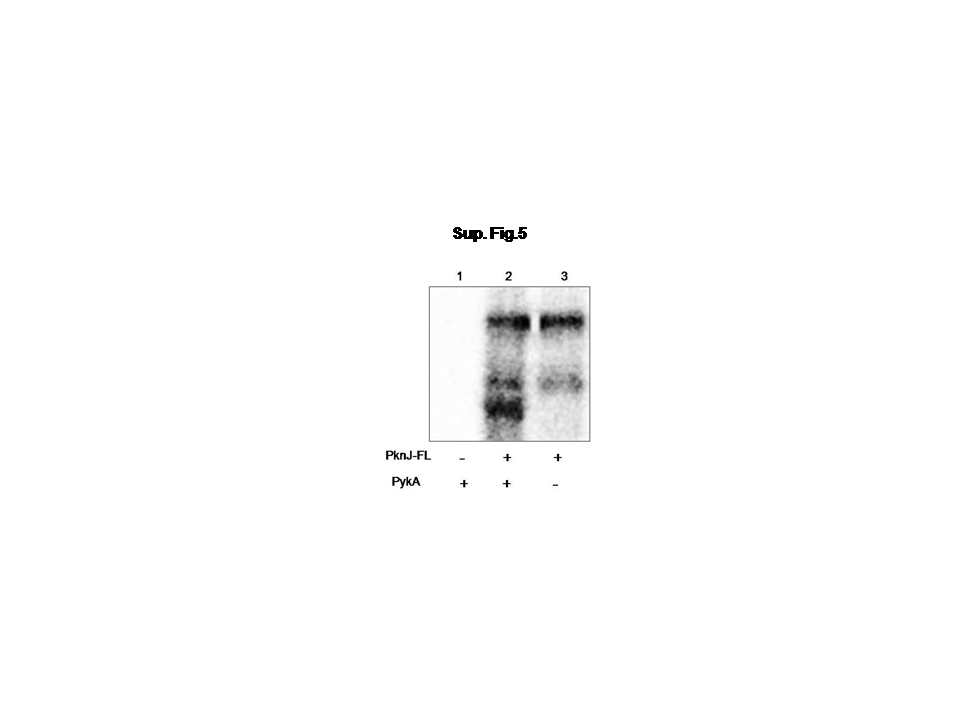

Supplement: Figure S5 — In vitro phosphorylation of mtPykA by PknJ-FL. 2 µg of kinase was incubated with 3 µg mtPykA. The reactions were run on 12% SDS-PAGE and gel was autoradiographed after drying. (2.79 MB TIF) [file pone.0010772.s005.tif]

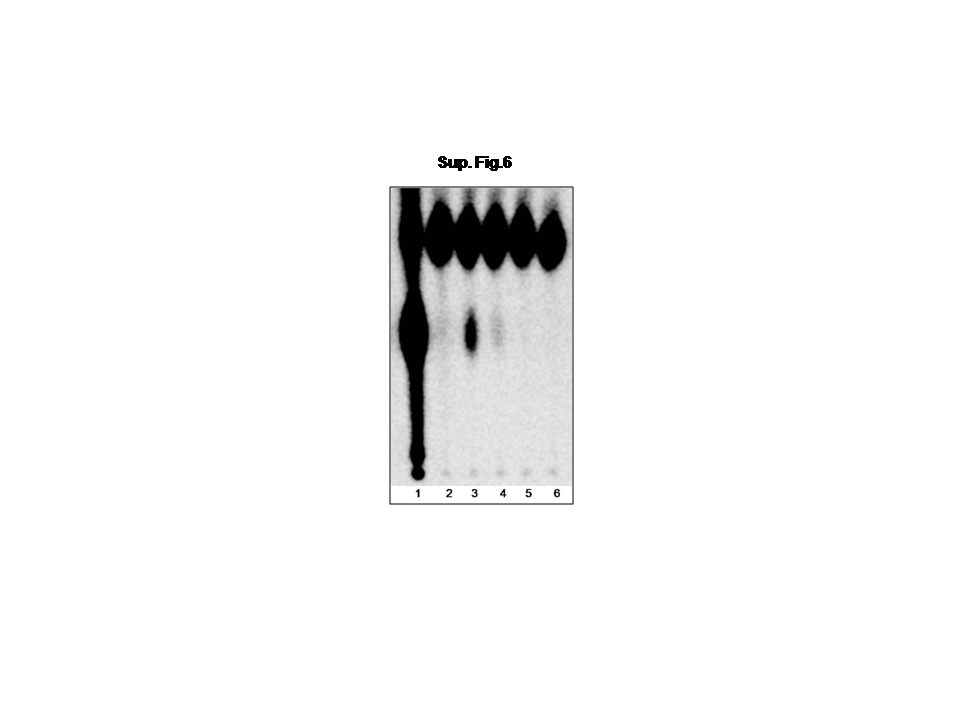

Supplement: Figure S6 — Activity assay of WT-PykA and PykA-S37A. Autoradiograph of cellulose-TLC is shown. Lane1: [α-32P]ATP +PEP + buffer control, Lane2: [α-32P]ADP + PEP+ buffer control, Lane3: [α-32P]ADP + PEP + WT-PykA, Lane4: [α-32P]ADP + PEP + PykA-S37A, Lane5: [α-32P]ADP + WT-PykA-PEP control, Lane6: [α-32P]ADP + PykA-S37A-PEP control. Loss of ATP generation is evident in case of PykA-S37A as compared to WT-PykA. (2.79 MB TIF) [file pone.0010772.s006.tif]
