# Supplementary material for: Understanding the Role of PknJ in Mycobacterium tuberculosis: Biochemical Characterization and Identification of Novel Substrate Pyruvate Kinase A
Source: PLoS One. 2010 May 24;5(5):e10772. doi: 10.1371/journal.pone.0010772 (PMC2875399; doi:10.1371/journal.pone.0010772)
Supplement: File S1 — List of previously reported selective substrates of M. tuberculosis STPK containing the putative phosphorylation motifs RXXXS/T, RXXS/T and RXS/T. (0.03 MB DOC) [file pone.0010772.s007.doc]

List of previously reported selective substrates of *M. tuberculosis* STPK containing the putative phosphorylation motifs RXXXS/T, RXXS/T and RXS/T.

> Rv2245

1 - VSQPSTANGG FPSVVVTAVT ATTSISPDIE STWKGLLAGE SGIHALEDEF VTKWDLAVKI

61 - GGHLKDPVDS HMGRLDM**RRM S**YVQRMGKLL GGQLWESAGS PEVDPDRFAV VVGTGLGGAE

121 - **RIVES**YDLMN AGGP**RKVS**PL AVQMIMPNGA AAVIGLQLGA RAGVMTPVSA CSSGSEAIAH

181 - AWRQIVMGDA DVAVCGGVEG PIEALPIAAF SMM**RAMS**TRN DEPE**RAS**RPF DKDRDGFVFG

241 - EAGALMLIET EEHAKARGAK PLARLLGAGI TSDAFHMVAP AADGVRAG**RA MT**RSLELAGL

301 - SPADIDHVNA HGTATPIGDA AEANAIRVAG CDQAAVYAPK SALGHSIGAV GALESVLTVL

361 - TLRDGVIPPT LNYETPDPEI DLDVVAGEPR YGDYRYAVNN SFGFGGHNVA LAFGRY

> Rv2246

1 - VGVPPLAGAS RTDMEGTFA**R PMT**ELVTGKA FPYVVVTGIA MTTALATDAE TTWKLLLD**RQ**

61 - **S**GIRTLDDPF VEEFDLPVRI GGHLLEEFDH QLTRIELRRM GYLQ**RMST**VL SRRLWENAGS

121 - PEVDTN**RLMV S**IGTGLGSAE ELVFSYDDMR ARGMKAVSPL TVQKYMPNGA AAAVGLERHA

181 - KAGVMTPVSA CASGAEAIAR AWQQIVLGEA DAAICGGVET RIEAVPIAGF AQM**RIVMS**TN

241 - NDDPAGACRP FDRDRDGFVF GEGGALLLIE TEEHAKARGA NILARIMGAS ITSDGFHMVA

301 - PDPNGERAGH AITRAIQLAG LAPGDIDHVN AHATGTQVGD LAEGRAINNA LGGNRPAVYA

361 - PKSALGHSVG AVGAVESILT VLALRDQVIP PTLNLVNLDP EIDLDVVAGE PRPGNYRYAI

421 - NNSFGFGGHN VAIAFGRY

> Rv1747

1 - VPMSQPAAPP VLTV**RYEGS**E RTFAAGHDVV VGRDLRADVR VAHPLISRAH LLLRFDQGRW

61 - VAIDNGSLNG LYLNNRRVPV VDIYDAQRVH IGNPDGPALD FEVG**RHRGS**A G**RPPQT**TSIR

121 - LPNLSAGAWP TDGPPQTGTL GSGQLQQLPP ATTRIPAAPP SGPQP**RYPT**G GQQLWPPSGP

181 - QRAPQIY**RPP T**AAPPPAGA**R GGT**EAGNLAT SMMKILRPG**R LT**GELPPGAV RIGRANDNDI

241 - VIPEVLAS**RH HAT**LVPTPGG TEI**RDNRS**IN GTFVNGARVD AALLHDGDVV TIGNIDLVFA

301 - DGTLARREEN LLETRVGGLD V**RGVT**WTIDG DKTLLDGISL TARPGMLTAV IGPSGAGKST

361 - LARLVAGYTH PTDGTVTFEG HNVHAEYASL RSRIGMVPQD DVVHGQLTVK HALMYAAELR

421 - LPPDTTKDDR TQVVARVLEE LEMSKHIDTR VDKLSGGQ**RK RAS**VALELLT GPSLLILDEP

481 - TSGLDPALD**R QVMT**MLRQLA DAGRVVLVVT HSLTYLDVCD QVLLLAPGGK TAFCGPPTQI

541 - GPVMGTTNWA DIFSTVADDP DAAKARYLA**R TGPT**PPPPPV EQPAELGDPA HTSLF**RQFST**

601 - IARRQLRLIV SDRGYFVFLA LLPFIMGALS MSVPGDVGFG FPNPMGDAPN EPGQILVLLN

661 - VGAVFMGTAL TIRDLIGERA IFRREQAVGL STTAYLIAKV CVYTVLAVVQ SAIVTVIVLV

721 - GKGGPTQGAV ALSKPDLELF VDVAVTCVAS AMLGLALSAI AKSNEQIMPL LVVAVMSQLV

781 - FSGGMIPVTG RVPLDQMSWV TPARWGFAAS AATVDLIKLV PGPLTPKDSH WHHTASAWWF

841 - DMAMLVALSV IYVGFVRWKI RLKAC

> Rv0019c

1 - MQGLVLQLTR AGFLMLLWVF IWSVL**RILKT** DIYAPTGAVM MRRGLAL**RGT** LLGARQRRHA

61 - ARYLVVTEGA LTGA**RITLS**E QPVLIG**RADD S**TLVLTDDYA STRHARLSM**R GS**EWYVEDLG

121 - STNGTYLD**RA KVT**TAVRVPI GTPV**RIGKT**A IELRP*

>Rv3223c

1 - MADIDGVTGS AGLQPGPSEE TDEELTARFE RDAIPLLDQL YGGAL**RMT**RN PADAEDLLQE

61 - TMVKAYAGFR SF**RHGT**NLKA WLY**RILT**NTY INSYRKKQRQ PAEYPTEQIT DWQLASNAEH

121 - SSTGLRSAEV EALEALPDTE IKEALQALPE EFRMAVYYAD VEGFPYKEIA EIMDTPIGTV

181 - MSRLHRGRRQ LRGLLADVAR DRGFARGEQA HEGVSS

> Rv3082c

1 - MELGSLI**RAT** NLWGYTDLMR ELGADPLPFL RRFDIPPGIE HQEDAFMSLA GFVRMLEASA

61 - AELDCPDFGL RLARWQGLGI LGPVAVIA**RN AAT**LFGGLEA IGRYLYVHSP ALTLTVSSTT

121 - ARSNVRFGYE VTEPGIPYPL QGYELSMANA ARMIRLLGGP QARA**RVFS**FR HAQLGTDAAY

181 - REALGCTV**RF GRT**WCGFEVD HRLAGRPIDH ADPETK**RIAT** KYLESQYLPS DATLSERVVG

241 - LAR**RLLPT**GQ CSAEAIADQL DMHPRTLQRR LAAEGLRCHD LIERERRAQA ARYLAQPGLY

301 - LSQIAVLLGY SEQSALNRSC RRWFGMTPRQ YRAYGGVSGR

> Rv2150c

1 - MTPPHNYLAV IKVVGIGGGG VNAVNRMIEQ GLKGVEFIAI NTDAQALLMS DADVKLDVG**R**

61 - **DST**RGLGAGA DPEVGRKAAE DAKDEIEELL RGADMVFVTA GEGGGTGTGG APVVASIARK

121 - LGALTVGVVT **RPFS**FEGKRR SNQAENGIAA L**RES**CDTLIV IPNDRLLQMG DAAVSLMDAF

181 - RSADEVLLNG VQGITDLITT PGLINVDFAD VKGIMSGAGT ALMGIGSARG EGRSLKAAEI

241 - AINSPLLEAS MEGAQGVLMS IAGGSDLGLF EINEAASLVQ DAAHPDANII FGTVIDDSLG

301 - DEV**RVT**VIAA GFDVSGPGRK PVMGETGGAH **RIES**AKAGKL TSTLFEPVDA VSVPLHTNGA

361 - TLSIGGDDDD VDVPPFMRR
